# An Ecological Study of a Universal Employee Depression Awareness and Stigma Reduction Intervention: “Right Direction”

**DOI:** 10.3389/fpsyt.2021.581876

**Published:** 2021-08-20

**Authors:** Benjamin Doty, Adrienne Grzenda, Seungyoung Hwang, Sean Godar, Darcy Gruttadaro, Kimberly A. Hauge, Bruce Sherman, Diana E. Clarke

**Affiliations:** ^1^American Psychiatric Association, Washington, DC, United States; ^2^Department of Psychiatry & Biobehavioral Sciences, David Geffen School of Medicine at UCLA, UCLA-Olive View Medical Center, Los Angeles, CA, United States; ^3^Department of Health Policy and Management, Johns Hopkins Bloomberg School of Public Health, Baltimore, MD, United States; ^4^Employers Health, Dublin, OH, United States; ^5^Human Resources, Kent State University, Kent, OH, United States; ^6^School of Medicine, Case Western University, Cleveland, OH, United States; ^7^Department of Mental Health, Johns Hopkins Bloomberg School of Public Health, Baltimore, MD, United States

**Keywords:** depression, anxiety, substance use disorders, wellness program, health care resource utilization, ecological study, employee depression awareness

## Abstract

**Objective:** Right Direction (RD) was a component of a universal employee wellness program implemented in 2014 at Kent State University (KSU) to increase employees' awareness of depression, reduce mental health stigma, and encourage help-seeking behaviors to promote mental health. We explored changes in mental health care utilization before and after implementation of RD.

**Methods:** KSU Human Resources census and service use data were used to identify the study cohort and examine the study objectives. A pre-post design was used to explore changes in mental health utilization among KSU employees before and after RD. Three post-intervention periods were examined. A generalized linear mixed model approach was used for logistic regression analysis between each outcome of interest and intervention period, adjusted by age and sex. Logit differences were calculated for post-intervention periods compared to the pre-intervention period.

**Results:** Compared to the pre-intervention period, the predicted proportion of employees seeking treatment for depression and anxiety increased in the first post-intervention period (OR = 2.14, 95% Confidence Interval [CI] = 1.37–3.34), then declined. Outpatient psychiatric treatment utilization increased significantly in the first two post-intervention periods (OR =1.89, 95% CI = 1.23–2.89; OR = 1.75, 95% CI = 1.11–2.76). No difference was noted in inpatient psychiatric treatment utilization across post-intervention periods. Unlike prescription for anxiolytic prescriptions, receipt of antidepressant prescriptions increased in the second (OR = 2.25, 95% CI = 1.56–3.27) and third (OR = 2.16, 95% CI = 1.46–3.20) post-intervention periods.

**Conclusions:** Effects of RD may be realized over the long-term with follow-up enhancements such as workshops/informational sessions on mindfulness, stress management, resiliency training, and self-acceptance.

## Introduction

Approximately one in five adults aged 18 and over in the United States (US) has a diagnosable mental disorder (1). The sequelae of mental disorders can impair the ability or motivation to work, contributing to absenteeism, increased cost to employer-sponsored health plans, and overall lower productivity (2–5). In recent years, employers and employees in the US are increasingly aware of the need to address mental health in the workplace, an important but underutilized venue for promoting wellness and increasing access to mental health services (6, 7). While feasible for employers and organizations to implement workplace-based mental health initiatives, there is a need to continue building the evidence base as to the impact of these interventions on mental health-related outcomes.

Mental health care utilization metrics, such as receipt of treatment, are valuable for quantifying the effects of workplace mental health initiatives. These data help demonstrate return on investment and support future service planning. Population-based surveys of healthcare utilization in the US show that a large proportion of adults with depressive disorders do not receive treatment. A study of the Medical Expenditure Panel Survey database found that while the proportion of depressed patients who receive antidepressant treatment has increased in recent decades, approximately one-third of adults diagnosed with major depressive disorder were not on any antidepressant treatment in 2015 (8). A study of the National Survey on Drug Use and Health found that approximately 34% of adults with major depressive episode received no treatment at all in 2019, and this treatment gap has remained steady since 2009 (9). While undertreated at the population level, depression is a leading cause of work disability and, thus, a chief driver of health and disability claims in many organizations (6, 10). From 2005 to 2010, the economic costs of major depressive disorder (MDD) increased by 21.5% with 50% of costs attributable to the workplace (9).

While many studies have been conducted on workplace-based mental health interventions, large-scale evidence is needed on the effectiveness of these initiatives for specific mental health outcomes, including mental health care utilization (11–13). From a public health perspective, universal interventions may be an effective approach for preventing mental illness or improving mental health in the workplace because organizations have existing channels to reach employees, enabling assessment of targeted outcomes (12). Such evidence helps demonstrate intervention value to stakeholders and justifies future organizational commitment to these initiatives. The current study aimed to explore differences in mental health utilization outcomes before and after implementation of a large-scale workplace intervention at Kent State University (KSU).

## Methods

### Intervention

KSU is a public research university located in Kent, Ohio that employs over 6,000 academic and administrative staff members. The KSU administration identified depression as one of the most burdensome mental disorders contributing to absenteeism and reduced productivity among its employees. In 2012, KSU launched a five-year universal employee wellness program (Wellness Your Way) that took a holistic approach to employee health management and promotion by focusing on personal well-being, work-life balance, and mental health. Wellness Your Way included Right Direction (RD), a depression awareness initiative developed by the American Psychiatric Association (APA) Foundation's Center for Workplace Mental Health and Employers Health. RD sought to increase depression awareness, reduce mental health stigma, and promote help-seeking behaviors. RD offers turnkey and customizable tools, resources, and guidance that employers can use to supplement existing employee assistance programs (EAP) and healthcare benefits.

A customized version of RD was implemented at KSU and rolled out in two phases from May through September 2014. In the first phase, 400 KSU managers and supervisors were: (1) provided with informational resources to recognize signs and symptoms of depression and how to support affected employees; and (2) informed about available services resources at KSU (e.g., EAP) for employees experiencing depression or other mental health problems by attending at least one of 36 educational or informational sessions held across KSU's eight campuses. Following phase one, the second phase of RD was rolled out to all 6,000 KSU employees through: (1) dissemination of promotional materials (e.g., informational posters and emails, monthly wellness newsletters), including the contact and website information for RD and the university's EAP; (2) ten open enrollment benefit fairs for employees, which included information to increase the visibility of available health services and resources at KSU; Following the initial implementation of RD in 2014, activities to promote and extend its reach were carried out in 2015 through 2017. For instance, enhancements and wellbeing activities (i.e., workshops and/or informational sessions for mindfulness, mindfulness meditation walks, yoga, stress management, resiliency training, and gratitude and self-acceptance) were included with RD and rolled out to the KSU employee population in 2016 and 2017.

### Data

An evaluation of RD at KSU was not planned *a priori*. However, after the intervention ended, secondary administrative data (i.e., KSU Human Resources (HR) census, insurance, and pharmacy claims data) were used to determine if RD affected the KSU employee population. The study involved secondary data analysis of limited data and did not involve direct employee contact; therefore, informed consent was not required. The research protocol received expedited review and approval from APA's Institutional Review Board. The HR census data contained information on 5,463 KSU employees who were actively employed at the institution on May 1, 2013–the target population for the study. A combination of generalization and suppression techniques using a k-anonymity privacy model of *k* = 5 (14), resulted in 36 employees being excluded from the target sample. In addition, those who terminated employment with KSU before the start of the RD program were excluded, leaving a final eligible population of 3,977 employees. The final dataset included sociodemographic information including age, gender, race, marital status, and employment status. International Classification of Diseases (ICD)-9-Clinical Modification (CM) codes (290–319) and associated ICD-10-CM codes for mental and substance use disorders were used to identify employees with any diagnosed mental or substance use disorders from Anthem and/or Medical Mutual claims data under university insurance benefits.

### Statistical Analysis

The outcomes of interest were changes in mental health utilization, specifically the changes in the estimated proportion of employees with insurance or pharmacy claims for: (1) treatment of depression or anxiety; (2) inpatient treatment for any psychiatric diagnosis; (3) outpatient treatment for any psychiatric diagnosis; (4) receipt of anxiolytic medications; or (5) receipt of antidepressant medications. We utilized a pretest-posttest design with 1 pre-intervention period and 3 post-interventions periods where each participant's pre-intervention (i.e., non-exposure) and post-intervention (i.e., exposure) periods were compared. The pretest period represented the 12 months (i.e., May 1, 2013–April 30, 2014) prior to the initiation of RD, which ran for 5 months (May 1–September 30, 2014). The posttest periods represented 1 to 12 (posttest 1: October 1, 2015–September 30, 2016), 13 to 24 (posttest 2: October 1, 2016–September 30, 2016), and 25 to 33 (posttest 3: October 1, 2016–June 30, 2017) months after the 5-month intervention period. These post-intervention periods were selected to demarcate relatively similar timeframes for comparison and to examine the potential sustainability of the effects of RD.

All statistical analyses were performed using SAS, version 9.4 (SAS Institute Inc., Cary, North Carolina) with alpha = 0.05 as the cutoff for determining statistical significance. Descriptive statistics were calculated for sociodemographic variables for each period. Differences between the pre-intervention group and each post-intervention group were evaluated by Wilcoxon signed-rank test for continuous variables and Chi-square test for categorical variables. A generalized linear mixed model (GLM) approach was used for logistic regression analysis between each outcome of interest and period, adjusting for age and sex (binomial model with logit link). Least squares means (LS-means), or the predicted population margins of the logits, were calculated for each period. Pairwise comparisons were made between logit estimates for all post-intervention periods to the pre-intervention period. Tukey-Kramer multiple comparison adjustment was made for the *p-*values for the differences of LS-means (difference in the logits). Differences are presented as LS-mean differences as well as odds ratios (exponentiation of the logit differences).

## Results

Table 1 shows the sociodemographic composition of the pre-intervention and post-period KSU employees. Attrition in this study was cumulative; those who left KSU were not sought at the later study period. These employees were more likely to be younger and to not report race or marital status (data not shown). Despite attrition, the sociodemographic composition of employees was comparable across all time periods except for race (*X*^2^ *P* = 0.0001), likely due to the high rate of missingness in this variable in the pre-intervention and first post-period. When missing values were excluded, the time periods demonstrated no differences in race distribution (*X*^2^ *P* = 0.96).

**Table 1:** Sociodemographic and diagnostic characteristics of KSU employees across study periods.

**Characteristic^a^**	**Pre-intervention** ***N* = 3,977**	**Post-period 1** ***N* = 3,547**	**Post-period 2** ***N* = 2,927**	**Post-period 3** ***N* = 2,492**
Age in 2017 (Mean, SD)	50.3 ± 12.0	50.6 ± 11.9	50.6 ± 11.6	50.8 ± 11.4
Female	2,255 (56.7)	2,005 (56.5)	1,645 (56.2)	1,382 (55.5)
Race^*^				
White	2,548 (64.1)	2,392 (67.4)	2,143 (73.2)	1,808 (72.6)
Black	391 (9.8)	353 (10.0)	304 (10.4)	269 (10.8)
Asian	368 (9.3)	348 (9.8)	328 (11.2)	296 (11.9)
Other or Missing	655 (16.5)	454 (12.8)	152 (5.2)	119 (4.8)
Marital status				
Married / Life Partner	2,295 (57.7)	2,065 (58.2)	1,712 (58.5)	1,481 (59.4)
Single	744 (18.7)	668 (18.8)	549 (18.8)	463 (18.6)
Divorced / Separated / Widowed	373 (9.4)	336 (9.5)	277 (9.5)	231 (9.3)
Other or Missing	565 (14.2)	478 (13.5)	389 (13.3)	317 (12.7)

a *Data are presented as raw frequency (percentages) unless otherwise indicated. Differences in distribution of variables across study periods were examined by Wilcoxon-rank sum test (continuous) or Chi-square test (categorical)*.

* *= Chi-square p-value < 0.0001*.

**Figure 1:**
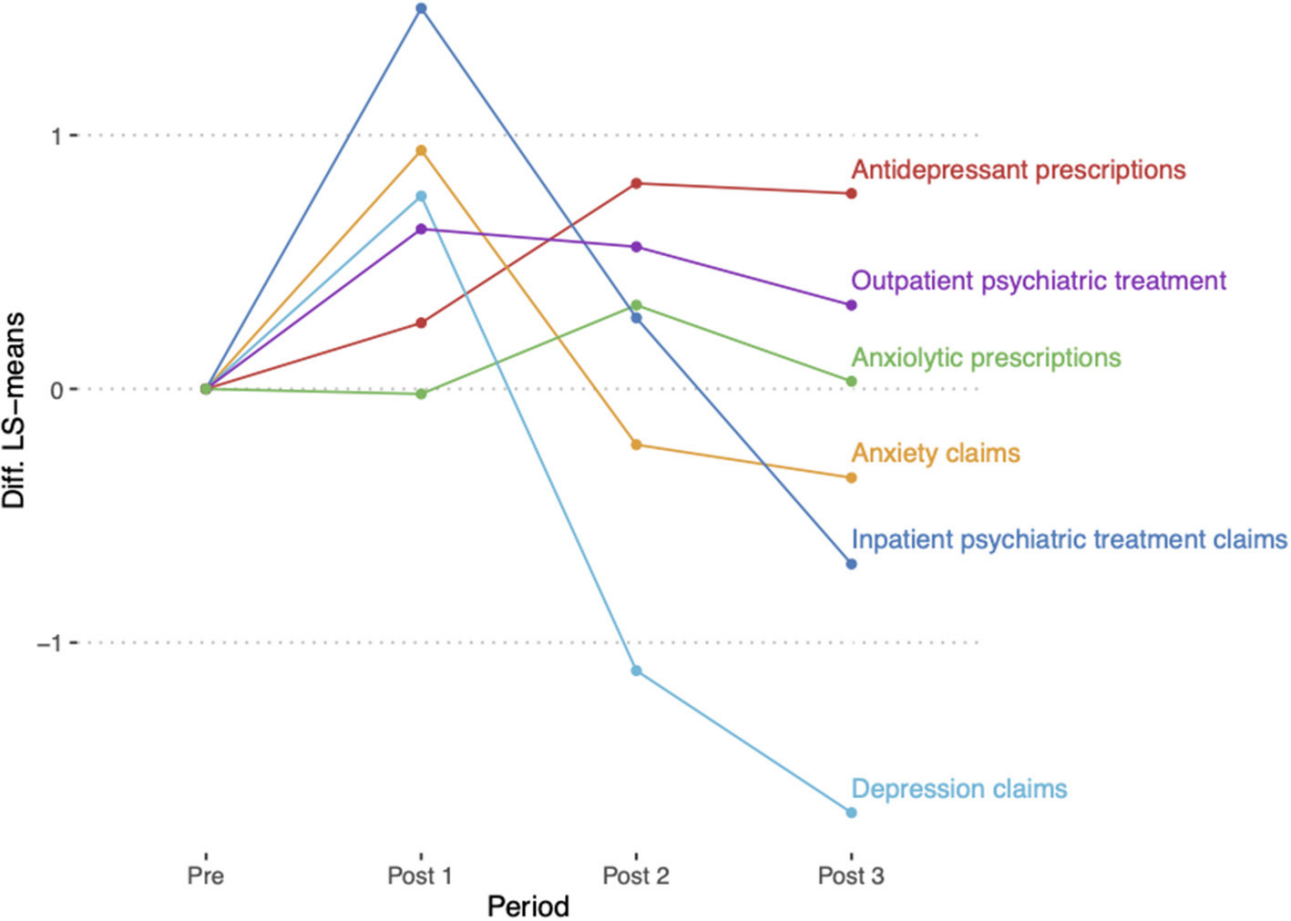
Comparison of mental health utilization between post- and pre-intervention periods. Differences in the least square means (LS-means, predicted population margins in logit scale) were calculated for each outcome for each post-intervention period compared to the pre-intervention period.

Table 2 and Figure 1 show the differences in predicted population margins for each outcome for each post-intervention period compared to the pre-intervention period. The difference in the proportion of employees seeking treatment for depression or anxiety increased in the first post-intervention period (+0.76, *P* < 0.0001 OR = 2.14; +0.94, *P* < 0.0001, OR = 2.55, respectively) but decreased in subsequent post-treatment periods. No significant differences were found for any post-intervention period in the proportion of employees with an inpatient hospitalization for treatment of any psychiatric illness. The proportion of employees with outpatient treatment for any mental health diagnosis was higher during post-intervention period 1 (+0.63, *P* = 0.0005, OR = 1.89) and 2 (+0.56, *P* = 0.008, OR = 1.75). The proportion of employees receiving antidepressant medications was higher during post-intervention period 2 (+0.81, *P* < 0.0001, OR = 2.25) and 3 (+0.77, *P* < 0.0001, OR = 2.16) compared to the pre-intervention period. No significant differences were found between the pre- and post-intervention periods for receipt of anxiolytic medications.

**Table 2:** Comparison of mental health utilization between post- and pre-intervention periods.

**Outcome**	**Contrast**	**Difference in LS-means (SE)^a^**	**OR (adj. 95% CI)^b^**	**Adj. *p*-value^c^**
Depression treatment	Post-period 1 vs. Pre-intervention	+0.76 (0.16)	2.14 (1.37–3.34)	<0.0001
	Post-period 2 vs. Pre-intervention	−1.11 (0.21)	0.33 (0.19–0.59)	<0.0001
	Post-period 3 vs. Pre-intervention	−1.67 (0.25)	0.20 (0.10–0.37)	<0.0001
Anxiety treatment	Post-period 1 vs. Pre-intervention	+0.94 (0.16)	2.55 (1.65–3.96)	<0.0001
	Post-period 2 vs. Pre-intervention	−0.22 (0.20)	0.81 (0.47–1.38)	0.81
	Post-period 3 vs. Pre-intervention	−0.35 (0.21)	0.71 (0.39–1.27)	0.49
Inpatient psychiatric treatment	Post-period 1 vs. Pre-intervention	+1.5 (0.68)	4.47 (0.69–29.92)	0.19
	Post-period 2 vs. Pre-intervention	+0.28 (0.85)	1.32 (0.13–13.3)	0.99
	Post-period 3 vs. Pre-intervention	−0.69 (1.19)	0.50 (0.02–12.77)	0.98
Outpatient psychiatric treatment	Post-period 1 vs. Pre-intervention	+0.63 (0.16)	1.89 (1.23–2.89)	0.0005
	Post-period 2 vs. Pre-intervention	+0.56 (0.17)	1.75 (1.11–2.76)	0.008
	Post-period 3 vs. Pre-intervention	+0.32 (0.18)	1.38 (0.84–2.27)	0.39
Antidepressant prescriptions	Post-period 1 vs. Pre-intervention	+0.26 (0.13)	1.29 (0.91–1.83)	0.26
	Post-period 2 vs. Pre-intervention	+0.81 (0.14)	2.25 (1.56–3.27)	<0.0001
	Post-period 3 vs. Pre-intervention	+0.77 (0.14)	2.16 (1.46–3.20)	<0.0001
Anxiolytic prescriptions	Post-period 1 vs. Pre-intervention	−0.02 (0.13)	0.98 (0.70–1.38)	0.99
	Post-period 2 vs. Pre-intervention	+0.33 (0.13)	1.40 (0.98–1.98)	0.07
	Post-period 3 vs. Pre-intervention	+0.03 (0.14)	1.03 (0.70–1.51)	0.99

a *Least squares means (LS-means) and standard errors (SE) were calculated from a generalized linear mixed model (e.g., binomial model with logit link) for each outcome and study period, adjusted by age and sex. Post-intervention periods were contrasted to the pre-intervention period, presented here as the differences in LS-means (logit scale)*.

b *OR = Odds ratio (Tukey-adjusted 95% Confidence Interval)*.

c *P-values were adjusted to account for multiple comparison testing by the Tukey method*.

## Discussion

In this study, we explored differences in mental health utilization by KSU employees following implementation of Right Direction—a depression awareness and stigma reduction intervention implemented at KSU in 2014. We found that the predicted margin of employees seeking treatment for depression and anxiety increased in the first post-intervention period but subsequently decreased in the other post-intervention periods. Employees seeking outpatient treatment for any mental health diagnosis increased during the first two post-intervention periods, then decreased. Receipt of antidepressant medication increased during post-intervention periods especially the second and third post-intervention periods.

This pattern would indicate that increased awareness of available resources resulted in an increased number of employees seeking assistance. The lag in receipt of antidepressant medication is anticipated. An employee seeking care would be likely to have more frequent visits while initiating or adjusting medications with the number of visits (e.g., claims) reducing as the individual enters remission or maintenance (also evident from the plateau in receipt of antidepressants). In addition, the modest increase or no change between pre-intervention and post-intervention periods with regards to anxiolytic prescriptions suggests patient were primarily prescribed antidepressants, which is the accepted first line treatment (15).

Given RD's focus on educational activities, we surmise that the short-term increase in employees using antidepressant and outpatient services may be attributable to greater employee awareness of the symptoms of depression and availability of mental health services. The lagged increased in receipt of antidepressant medications and reduction in the number of claims for depression treatment may indicate maintenance management of these individuals since there were no modifications in KSU health plan design. However, this also suggests that the effects of RD were short-lived and one implementation of RD without enhancements may be insufficient to significantly change the proportion of employees seeking care over time at the institutional population level. A systematic review of universal workplace interventions on depressive symptoms found overall positive but small effects, but a challenge for these approaches is resource and time allocation to sufficiently engage the target population, especially in broad organizational level approaches which are less frequently studied (11). An investigation of a variety of workplace wellness programs implemented in more than 300 businesses in the US found higher overall employee participation rates in small organizations (<500 employees) than in large organizations (>500 employees), perhaps because employee engagement is less administratively complex or more compelling in small networks of employees (16). Other drivers of employee engagement are organizational health norms and sustained organizational efforts to create a culture of employee health (17). While KSU employees' level of participation in RD activities is unknown, mental health engagement was a focal point in the university's employee health strategy, and the present study's findings suggest that the effects of broad organizational level approaches deserve further attention.

The present study's findings also have implications for the practice of employee mental health management. First, employee wellness officials implementing RD at their institution could anticipate that the effects of the program may be realized slowly, given that individual health behavior change tends to happen gradually (18, 19). Officials implementing RD in the future should consider long-term monitoring of program effects. Second, planning follow-up enhancements after initial RD program implementation may be helpful. In complex organizations, a guiding principle for achieving change is to aim for incremental improvements within a comprehensive strategy (20). As the KSU implementation illustrates, implementing RD at large institutions could entail a long-term, tailored communications strategy accompanied by frequent and consistent workshops and informational sessions on topics such as mindfulness, meditation, stress management, resiliency training, and gratitude and self-acceptance (21). A recent meta-analysis of 57 studies of message- and material-focused behavior change interventions found that program tailoring (e.g., to the target population and context), number of intervention contacts, and length of follow-up, among other factors, all significantly moderated intervention effects (22). Dedicated staff (i.e., “program champions”) were essential to the sustained, tailored roll out of RD at KSU, and prior research also points to the benefits of training employers on best practices for designing, implementing, and evaluating workplace health programs (23).

This study had several limitations. This was an ecological analysis done several years after RD was implemented. We did not have information on the degree of exposure to the intervention that individual KSU employees received. RD at KSU was implemented as a multi-component intervention, and the data do not enable an analysis to tease apart the effects of the various components. As our analyses were only adjusted for age and sex, the possibility of uncontrolled confounding cannot be ruled out. Evaluating the effects of workplace-based interventions is complex, as there are many individual, organizational, and societal factors that affect employee mental health (24, 25). It is possible that a secular trend or other macro-level phenomena may explain the findings. Recent studies suggest that mental health awareness and attitudes toward mental illness are slowly improving in the population (26). Other macro-level phenomena, such as changes in economic climate or university policies, could also explain the findings. However, the multiple pre-post comparisons over multiple years in this study somewhat mitigate these concerns. These methodological challenges highlight the importance of designing interventions and planning for evaluation concurrently. The employees of KSU are not likely to be representative of the general adult working population in the US, so the findings may not generalize to other sectors, such as private industry, or specific occupational groups not typically employed by a university.

In summary, we were able to link census data with medical and pharmacy claims data to explore mental health utilization outcomes following exposure to this workplace intervention, using multiple follow-up periods to better understand the long-term effects of RD and other follow-up enhancements.

## Conclusion

Employers are becoming increasingly cognizant of employee mental health. Turnkey workplace mental health programs, such as RD, offer a customizable approach for employers to promote employee mental health without straining organizational resources. Employers can use RD as a stand-alone or as a supplemental program to augment existing initiatives, such as EAPs and other mental health benefits. Increased employee awareness of available resources and services may result in increased employee care-seeking and engagement in care over time. The intended effects of the RD intervention may be observable in the long-term, and follow-up enhancements after initial implementation could be beneficial.

## Data Availability Statement

The data analyzed in this study is subject to the following licenses/restrictions. The datasets generated for this study will not be made publicly available. The data license agreement restricts the access only to the American Psychiatric Association. Requests to access these datasets should be directed to dclarke@psych.org.

## Ethics Statement

The study was approved by the American Psychiatric Association (APA) Institutional Review Board. The APA IRB and the study team took steps to ensure the study was not unduly influenced by the sponsor, Takeda Pharmaceuticals U.S.A., Inc. (TPUSA). The study was a secondary data analysis of data collected during routine service use by KSU employees. The study protocol, data, and analytic methods were approved by the APA IRB, whose members are not employed by APA and do not receive remuneration for their role on the IRB. Data collection, data analysis, and the interpretation of results were conducted independently of TPUSA. Written informed consent for participation was not required for this study in accordance with the national legislation and the institutional requirements.

## Author Contributions

DC and SG designed the study. KH collected the data. AG, SH, BD, and DC analyzed the data. All authors contributed to the writing of the manuscript. All authors reviewed and edited the final version of the manuscript. All authors read and approved the final manuscript.

## Conflict of Interest

The Center for Workplace Mental Health, a program of the American Psychiatric Association Foundation, also receives funding support for its work from Sunovion, Janssen, Otsuka Pharmaceutical, and Myriad. SG has received funding support from Jansson Pharmaceuticals for a research study in conjunction with Tufts University. The funding had no impact on the current research. BS discloses advisory board compensation from, Amgen, Regeneron and Medtronic; is a consultant to the National Alliance of Healthcare Purchaser Coalitions and Employers Health Coalition, Inc.; has received research grants from Takeda/Lundbeck, Pfizer, Sanofi, and the National Pharmaceutical Council; and is a speaker/received honoraria from IBM Watson, Pfizer, Merck, and AbbVie. DC served on the advisory panel for the RAND Corporation Mental Health Landscape project, which was funded by Otsuka Pharmaceuticals. The remaining authors declare that the research was conducted in the absence of any commercial or financial relationships that could be construed as a potential conflict of interest.

## Publisher's Note

All claims expressed in this article are solely those of the authors and do not necessarily represent those of their affiliated organizations, or those of the publisher, the editors and the reviewers. Any product that may be evaluated in this article, or claim that may be made by its manufacturer, is not guaranteed or endorsed by the publisher.
